# Response to the Editor: The Essential Component of Clinical Trials' Reports

**DOI:** 10.5812/nms.13833

**Published:** 2013-09-15

**Authors:** Manyat Ruchiwit

**Affiliations:** 1 Department of Mental Health and Psychiatric Nursing, Faculty of Nursing, Thammasat University, Klong Luang, Thailand

**Keywords:** Quasi-experimental research, Randomized block design, Matched pairs, Differences, Extraneous variables

Dear Editor,

Referring to the letter you sent to me, I would like to explain more about the research study and its results, “Effects of a three-stage intervention program on the holistic health status of patients with drug addiction after discharge”. I do understand that a well-designed experiment includes design features that allow researchers to eliminate extraneous variables as an explanation of the observed relationship between the independent variable(s) and the dependent variable.

In this study, the research design was a quasi-experimental research and a matched pairs design, which is a special case of the randomized block design. It is used when the experiment has only two treatment conditions (control and experimental); and participants can be grouped into pairs, based on some blocking variables. For example, in this study, the researcher manipulated one dependent variable, holistic health status, while holding all other extraneous variables constant. Therefore, the control variable was: being discharged from the institution within six months, and matching variables were: gender, age, level of education, and type of drug addiction, which might have affected the holistic status of the subjects according to the literature reviews (1 - 6). Then, within each pair, subjects were randomly assigned to different treatments for the experimental and the control groups. As with other designs, the matched pairs design uses randomization to control for confounding variables, and the sample size (N) of each group in this study was 45 which is larger than 30. However, it should be noted that, unlike the others, this design explicitly controlled for those extraneous variables that might have occurred, such as being discharged from the institution within 6 months, gender, age, level of education, and type of drug addiction, which were the differences in the baseline variables in the case of no control (Table 1). 

**Table 1.** Socio-demographic Characteristics of the Study Samples

Variables	Case, No.(%)	Control, No. (%)
**Gender**		
Male	17 (37.8)	17 (37.8)
Female	28 (62.2)	28 (62.2)
**Age, y**		
15-25	13 (28.9)	13 (28.9)
26-40	26 (57.8)	26 (57.8)
41-60	6 (13.3)	6 (13.3)
**Level of education**		
Primary school	16 (35.6)	16 (35.6)
Secondary School	24 (53.3)	24 (53.3)
University education	5 (11.1)	5 (11.1)
**Type of drug addiction**		
Depressants	12 (26.7)	12 (26.7)
Stimulants	31 (68.9)	31 (68.9)
Hallucinogens	2 (4.4)	2 (4.4)

After the experiment, a t-test was applied to see how the change occurred when compared between the experimental and control groups. Unlike the other designs, a significant level of change was tested, and therefore, a pre-test for baseline was not necessary due to the matched pairs being applied, and the differences in baseline variables were already controlled. In addition, this study tested the change (the differences, D1 and D2 of the experiment and control groups) because it is believed that this method helps to protect against the differences that might have been caused by the history and maturity of the subjects in the pre-post test of both experimental and control groups (7, 8).
